# Effects of Inulin-Based Prebiotics Alone or in Combination with Probiotics on Human Gut Microbiota and Markers of Immune System: A Randomized, Double-Blind, Placebo-Controlled Study in Healthy Subjects

**DOI:** 10.3390/microorganisms10061256

**Published:** 2022-06-20

**Authors:** Alessandra De Giani, Anna Sandionigi, Jessica Zampolli, Angela Michelotti, Francesco Tursi, Massimo Labra, Patrizia Di Gennaro

**Affiliations:** 1 Department of Biotechnology and Biosciences, University of Milano-Bicocca, Piazza Della Scienza 2, 20126 Milano, MI, Italy; alessandra.degiani@unimib.it (A.D.G.); anna.sandionigi@unimib.it (A.S.); jessica.zampolli@unimib.it (J.Z.); massimo.labra@unimib.it (M.L.); 2 Complife Italia S.r.l., Via Angelini 21, 27028 San Martino Siccomario, PV, Italy; angela.michelotti@complifegroup.com (A.M.); francesco.tursi@complifegroup.com (F.T.)

**Keywords:** synbiotic, *Lactobacillus*, *Bifidobacterium*, healthy subjects, gut microbiota, clinical study, immunological parameters, common infectious disease incidence

## Abstract

The gut microbiota is implicated in diverse interactions affecting human health. The present study reports a randomized, double-blind, placebo-controlled clinical study conducted by administering a new synbiotic formulation composed of two *Lactobacillus* strains (*L*. *plantarum* and *L*. *acidophilus*) and one *Bifidobacterium* strain (*B*. *animalis* subsp. *lactis*) and two types of fructans (fructo-oligosaccharides with a degree of polymerization of 3–5 and inulin-type fructans with 10 DP). The effects of this synbiotic were evaluated on healthy subjects for 28 days and the maintenance of its efficacy was evaluated at the end of a follow-up period of 28 days. The synbiotic treatment contributes to higher biodiversity of the gut microbiota, increasing the community richness with respect to the group with the prebiotics alone and the placebo group. Its positive effect is also reflected in the variation of microbial community structure favoring the beneficial short-chain fatty acids bacterial producers. The amelioration of the health status of the subjects was also established by the reduction of common infectious disease symptom incidence, the stimulation of the gut immune system showing a noteworthy variation of fecal β-defensin2 and calprotectin levels, and the modulation of the response of the respiratory tract’s immune system by salivary IgA as well as total antioxidant capacity biomarkers.

## 1. Introduction

The central district of human beings is represented by the gastrointestinal tract inhabited by the microbial communities playing a fundamental role in many physiological processes, such as absorption of nutrients, protection against ingested pathogens, and the maintenance of good health [1,2] also through the communication with the brain [3]. It is estimated that in the colon the population of microbes could reach up to 10^12^ colony forming units per gram of luminal content. Linked to microbial diversity, a variety of metabolic capabilities are associated with the gastrointestinal tract [4]. The gut microbiota has an important role in the digestion of food (such as fibers) and in the synthesis of vitamins and amino acids [5]. Furthermore, it covers crucial functions in supporting the immune system efficiency and in the modulation of host physiology and metabolism [6].

However, gut microbiota together with gut functions changes during a lifetime [7]. To date, elderliness is considered the last phase of life, usually associated with deterioration. In this respect, a universal definition of the elderly population is not available, because aging depends on biological, psychological, social, and environmental components. For instance, according to the United Nations report of World Ageing [8], people who are almost 60 years old can be considered elderly people. Age-related decline implies some alterations in the host physiology, in the immune system reactivity, as well as in the intestinal microbiota composition [9,10]. Nevertheless, the total number of bacteria remains quite constant in fecal samples of the elderly comparing to young people. Regarding the phyla that characterize the human gut microbiota, the most abundant are Firmicutes, Bacteroidetes, Actinobacteria, Proteobacteria, Verrucomicrobia, and Fusobacteria [6]. However, the intestinal microbiota is a very complex ecosystem [11], whose members play an important role despite the interpersonal variability [12]. Considering elderly people, some studies underlined that bifidobacteria decline [5], and others showed a reduction of *Bacteroides*, *Clostridia*, and Lactobacilli populations [7]. Likewise, the mucosal membranes covering the gastrointestinal tract are continuously exposed to pathogenic microorganisms and the consequent immune system response suffers a progressive age-related decline. This immune-senescence phenomenon can be also evaluated by different biological markers [13]. For example, fecal calprotectin and β-defensin2 are promising markers of the activation level of the immune system caused by bacterial metabolites significantly changing with age [14]. Moreover, immune system senescence can be also assessed at the respiratory tract level using among the other indicators, IgAs, which contribute to the gut microbiota homeostasis and have a crucial role against pathogens [15]. In addition, the total antioxidant capacity (TAC) can be also used as a biomarker for oxidative stress [16].

It is universally accepted that healthy gut microbiota contributes to an overall state of well-being which is crucial during the process of aging. In this context, it is considered worthwhile that an enrichment in the bacterial population that can ferment dietary fibers can be beneficial [12]. Therefore, a growing interest in adapted diets and the design of new formulations composed of selected probiotics, prebiotics, or synbiotics is emerging. Several molecules from natural sources like inulin and fructo-oligosaccharides (FOS) are the most studied prebiotics as well as resistant starch (RS), galacto-oligosaccharides (GOS), and xylo-oligosaccharides (XOS) [5]; furthermore, diverse papers highlight that dietary fibers contribute to the intestinal microbiota modulation, especially acting on the dominant species, indicating that a diversified microbiota could be favorable for the host [10,17]. For this reason, several studies evidence the role of synbiotic formulations as nutritional supplements containing living bacteria, i.e., *Bifidobacterium* strains [12], and fibers that could also be selectively utilized by host microorganisms leading to a gainful interaction with the resident intestinal microbiota [18,19,20,21]. In this respect, host gut microorganisms comprise both resident or autochthonous microorganisms and allochthonous microorganisms that can be externally applied (i.e., probiotics) and transiently members of the microbiota. Therefore, they can modulate the intestinal ecosystems together and thus ameliorate the function of the gut barrier and the immune response [18]. However, few papers report beneficial effects in terms of changes of metabolism and composition of gut microbiota and immune parameters in elderly people after synbiotic administration, and none of them reports the effect of either the prebiotic or the probiotic alone with respect to the synbiotic effect [12]. The majority describes that the effect could be influenced by the selected probiotic strains included in the formulate, and by the kind of selected prebiotics, in some cases also associated with a dose-response effect [4,22,23]. Only the study of Macfarlane et al. and Ouwehand et al. [18,19] on healthy elderly subjects reported the improvement of some markers of the intestinal microbiota composition associated with gastrointestinal tract functions (e.g., an increase of butyrate production deriving from the increase of Actinobacteria and Firmicutes and a reduction of the pro-inflammatory response) after the intake of different synbiotic formulations.

Considering all these aspects, it is very important to collect more data regarding the effectiveness of bacteria genera among probiotics (for example *Bifidobacterium* and *Lactobacillus*) and the efficacy related to the fibers (with respect to the synbiotics), and the correlation between the gut microbiota composition and the health status of elderly people [24].

In this context, the aim of the present study is the evaluation of the effectiveness of a new synbiotic formulation on both the composition of the gut microbiota and some biomarkers of the immune system in elderly subjects. Therefore, a double-blind, randomized, placebo-controlled clinical study was carried out by the administration of the formulation composed of two *Lactobacillus* strains (*L. plantarum* PBS067 and *L. acidophilus* PBS066), one *Bifidobacterium* strain (*B. animalis* spp. *lactis* BL050), and two types of fructans (fructo-oligosaccharides with a degree of polymerization 3–5 and inulin-type fructans with 10 DP). The treatment was performed in 28 days (T28) followed by a follow-up period of 28 days (T56), evaluating the gut microbial composition and modulation, the variability of fecal parameters including β-defensin2 and calprotectin, and salivary IgA, salivary total antioxidant capacity (TAC), and common infectious disease incidence (CID) as markers of well-being condition of all elderly subjects. This study design allowed us to assess the efficacy of the synbiotic treatment with respect to the group with the prebiotics alone and with respect to the placebo group.

## 2. Materials and Methods

### 2.1. Study Design

In line with the Declaration of Helsinki and the Good Clinical Practice guideline E6, a randomized, double-blind, placebo-controlled study was approved by the “Independent Ethical committee for Non-Pharmacological Clinical studies” and developed at Complife Italia S.r.l. facilities (Voghera, Italy).

All the enrolled subjects signed the informed consent form before the beginning of the study process. The study provided 75 subjects divided into three groups (A, B, D), randomly assigned to receive one mixture of prebiotic formulation (A) or synbiotic formulation (B) or placebo (D) once daily (the first day was T0) for 28 days (T28); then the subjects attended 28 days of post intervention period (28 days from the last ingestion of the tested products) (follow-up period) (T56).

The tested formulations consisted of food supplements in form of sticks (Farcoderma S.r.l., Torre Pallavicina (BG), Italy) containing *Lactobacillus* and *Bifidobacterium* probiotic strains [25,26] and/or prebiotics (Tables S1 and S2) as follows:

A. Prebiotic formulation (Group 1): 50 mg FOS (fructo-oligosaccharides with a degree of polymerization between 3–5) and 50 mg inulin (with a degree of polymerization of 10) (for a total of 100 mg of fibers), and common excipients used in formulations
B. Probiotic and prebiotic (synbiotic) formulation (Group 2): 1 × 10^9^ CFU *L. acidophilus* PBS066 (30 mg as lyophilized), 1 × 10^9^ CFU *L. plantarum* PBS067 (12 mg as lyophilized), 1 × 10^9^ CFU *B. animalis* spp. *lactis* BL050 (30 mg as lyophilized) (all the strains are from the private collection of Roelmi HPC, Origgio, Italy), with 50 mg FOS (fructo-oligosaccharides with a degree of polymerization between 3–5) and 50 mg inulin (with a degree of polymerization of 10) (for a total of 100 mg of fibers), and common excipients used in formulations
D. Placebo formulation (Group 3): the same formulation without prebiotics and probiotics included in the other two formulations.

**Figure 1:**
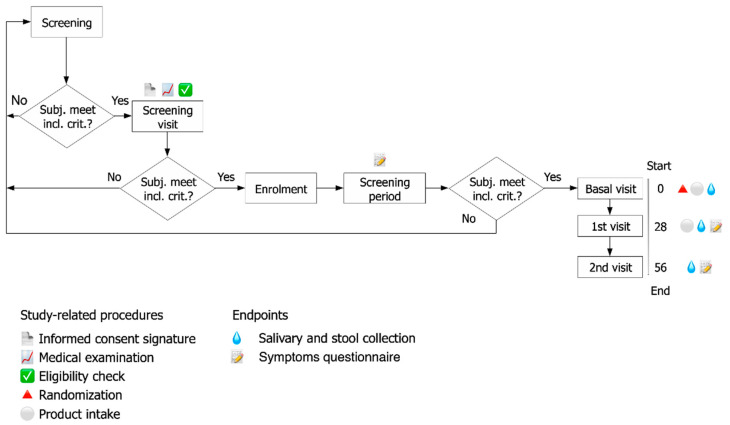
Study flow and schedule of assessment chart depicting the overall study design. The study corresponds to a randomized, double-blind, three-arm parallel-group, placebo-controlled. Levels of several immunological markers (fecal Calprotectin and ß-Defensin2; salivary IgA and total antioxidant capacity) and composition of intestinal microbiota (qPCR and NGS sequencing) were evaluated at three time-points during the study (baseline T0, at the end of the treatment period T28, and at the end of the follow-up T56).

The progressive steps of the study and the duration of each phase are outlined in the flow chart of Figure 1. Indications provided to the participants included the schedule of food supplement ingestion (away from meals, in a glass of non-sparkling water, once a day); the related protocol procedure; and a symptoms questionnaire.

Each subject collected fecal samples (at least 4 g) in a sterile plastic feces container at home and kept them at 4 °C for a maximum of 3 h until reaching the laboratory, where fecal samples were frozen immediately at −80 °C. An aliquot of 1 g was collected into Stool Nucleic Acid Collection and Preservation Tubes (Norgen Biotek Corp., Thorold, ON, Canada) until the subsequent analyses.

Salivary samples were collected directly at the medical center into sterile plastic tubes forbidding each subject to eat, drink, smoke tobacco-based products, or chew gum for minimum 30 min before the sampling.

Salivary samples for immunological parameters (IgA level and Total Antioxidant Capacity) and stool samples for fecal microbiota analysis and immunological parameters (Calprotectin and β-defensin2) were obtained at the starting point (T0) and after 28 days of formulate administration (T28) followed by sampling after the follow-up period of 28 days (T56).

The study was deposited as a clinical study with the following registration number: ISRCTN37538805.

### 2.2. Subjects of the Study

Eligible subjects were healthy elderly Caucasian males and females enrolled according to specific inclusion/exclusion criteria. Inclusion criteria were: (i) healthy free-living both gender elderly aged between 60 to 80 years old, with 18.5–24.99 Body Mass Index (BMI) on the day of inclusion, (ii) compliance with the signed informed consent form and trial procedures, (iii) vaccination against influenza, (iv) not modifying the daily routine comprising lifestyle and fitness, (v) not changing the usual food and fluid consumption and taking the proposed dietary supplement for all the study period, (vi) not using products that likely interfere with the testing formulations and using only the testing product during the whole study period.

Criteria for exclusion were: (i) contraindications to influenza vaccine, (ii) treatment for immune system modulation underwent in the previous 4 weeks, or immune-suppressant therapy underwent for more than 2 weeks or interrupted less than 3 months before the beginning of the study, (iii) inoculation with influenza vaccination less than one year before the study, (iv) being under antibiotic treatment, (v) affected by chronic diseases or having a familiarity to chronic conditions including congenital heart disease, liver or kidney disease, or immune deficiency, (vi) have received a probiotic treatment in the previous 6 months, (vii) ingestion of fiber products within the last 6 weeks, (viii) being affected by severe concurrent diseases, or (ix) abuse of drugs or alcohol.

### 2.3. Randomization

Randomization was performed according to Mezzasalma et al. [27] using PASS 11 statistical software (version 11.0.8 for Windows; PASS, LLC, Kaysville, UT, USA), with “Efron’s biased coin” algorithm. Subjects were randomized in a 1:1:1 (A, B, D) ratio. The resultant document was kept in a safe place in the study director due to double-blind restriction towards subjects, investigators, and collaborators.

### 2.4. Assessment of Clinical Effects

#### 2.4.1. Endpoints

The study was characterized by three main phases: the beginning of the study (baseline); the intake period, 28 days from the starting point to evaluate the effectiveness of the treatments measured as the recorded common infectious disease (CID) symptoms, the level of fecal calprotectin, fecal β-defensin2, salivary IgA, salivary total antioxidant capacity (TAC), and the composition of intestinal microbiota; and 28 days after the last formulation intake (follow-up) to assess the maintenance of the obtained effects.

#### 2.4.2. Symptoms Questionnaire

A questionnaire on general well-being and bowel habit was completed daily by each subject. The results were classified by the obtained bowel habit score varying from −3 (extreme decrease in the frequency of bowel movements) to +3 (increase in bowel movements), and 0 identifying a normal frequency [27].

Common infectious disease (CID) symptoms of subjects were also recorded through a questionnaire evaluating the status of GI and upper/lower respiratory tract [28]. Considered symptoms were graded on a 4-point-scale (from 0: no symptom to 3: severe symptom) and they included cough, hoarseness, sore throat, itchy throat, rhinorrhea, sneezing, nasal obstructions, fatigue, headache, myalgia, nausea, vomiting, and diarrhea. Then, each questionnaire was reviewed by the responsible doctor to verify the CID diagnosis. All the subjects were included in the calculation of the mean number of days with CID and the CID frequency, where no CID symptoms was reported as 0 value.

At each endpoint, a visit also verified the subject compliance to formulation consumption with respect to the study protocol. Compliance was satisfactory up to missing 2 doses during the 28 days of product intake, and each individual result did not exceed such limit.

#### 2.4.3. Assessment of Fecal Calprotectin

Calprotectin is a cytosolic calcium-binding protein that can be considered neutrophil-specific [29]. Calprotectin determination in feces provides a no invasive quantitative measure of neutrophil flux to the intestine and so can be considered as a marker of intestinal inflammation. Fecal calprotectin was measured using the PhiCal ELISA kit (NovaTec Immunodiagnostica GmBH, Dietzenbach, Germany) [29]. A calibration curve was used to quantify fecal calprotectin concentration with 2 ng/mL detection limit.

Calprotectin values minor than 45 μg/g stool were considered as normal values [29].

#### 2.4.4. Assessment of Fecal β-Defensin2

Fecal β-defensin2 (HβD-2) was quantified through ELISA Immunodiagnostik AG kit (Bensheim, Germany). The declared detection limit was 0.01 ng/mL [30,31].

#### 2.4.5. Assessment of Salivary IgA

The collected saliva samples were immediately centrifuged (at 5000× *g* for 15 min) after the sampling and subsequently the correspondent supernatants were stored at −20 °C as reported by Lefevre et al. [28]. Salivary IgA was quantified by ELISA test (Elabscience, Houston, TX, USA) detecting from 1.56 to 100 ng/mL and diluting accordingly to requirements.

#### 2.4.6. Assessment of Total Antioxidant Capacity (TAC) in Salivary Samples through FRAP

The antioxidant potential of salivary samples was measured by Ferric Reducing Antioxidant Parameter (FRAP) assay [32]. A standard curve of Fe(II) solutions was obtained measuring the absorbance values at 595 nm after 4 min of incubation at room temperature. Each sample was measured with the same procedure and the correspondent results were expressed as Fe(II) μM [33].

### 2.5. Probiotic Strains and DNA Extraction

The synbiotic formulation was set up using probiotic bacteria including two *Lactobacillus* spp. strains and one *Bifidobacterium* strain, supplied by Roelmi HPC (Origgio, Italy) private strain collection. Table S1 describes the characteristics of each strain, in terms of antimicrobial activity and growth capacity on the selected FOS and inulin-type compounds.

Microbial cultures were normally cultivated in De Man, Rogosa and Sharp medium (MRS) (Conda Lab, Madrid, Spain) and incubated at 37 °C for 24 h under anaerobic conditions obtained by anaerobic atmosphere generation bags for microbiology (Sigma-Aldrich, Milan, Italy). *B. animalis* spp. *lactis* culture was supplemented with 0.3 g/L L-cysteine hydrochloride monohydrate (Sigma-Aldrich, Italy).

Total DNA was extracted from each pure probiotic culture (with a title of 10^9^ CFU/mL) using Ultraclean Microbial DNA Isolation Kit (MoBIO Laboratories, Milan, Italy) to obtain standard curves useful to quantify the presence of these probiotics in stool samples by qPCR analyses using ten-fold dilutions ranging from 10^8^ CFU/mL to 10 CFU/mL [27].

Total DNA was extracted from 400 µL of stool samples conserved in preservation tubes, using Stool Nucleic Acid Isolation Kit (Norgen Biotek Corp., Thorold, ON, Canada) following the protocol provided by the manufacturer with some modifications [34]. Briefly, 400 µL of the sample dissolved in the preservation buffer were added to 600 µL of Lysis Buffer, vortexed for 5 min, and centrifuged for 4 min at room temperature. For the final elution of the nucleic acid sample, 50 µL and then other 50 µL (total volume of 100 µL of final sample) of Elution Buffer were used.

DNA from stool samples was also used for the microbiome sequencing through Illumina MiSeq platform [35].

### 2.6. Fecal Microbiology Analysis by Quantitative PCR

qPCR reactions were conducted using PCR Real-Time StepOne^TM^ Plus (Applied Biosystems, Monza, Italy) and the PowerUp^TM^ SYBR^TM^ Green Master Mix (Applied Biosystems, Monza, Itlay). The species-specific primers employed in this study were previously designed [27,36] and they are listed in Table S3. The amplification reactions were performed in a total volume of 10 µL, including 5.6 µL of PowerUp^TM^ SYBR^TM^ Green Master Mix, 10 µM Forward and Reverse primers, and 4.4 µL of DNA template. The qPCR program consisted of a first step of 10 min at 95 °C, then 40 cycles of 15 s at 95 °C and 1 min at 60 °C, followed by a melting curve [36].

All the employed DNA for the quantification deriving from pure microbial cultures or from stool samples were analyzed in triplicate.

Data on the quantification were expressed as mean values ± SEM. The average slope and *y*-intercept of each standard curve of *L. plantarum* PBS067, *L. acidophilus* PBS066, and *B. animalis* spp. *lactis* BL050 were determined by regression analysis and used to calculate the fecal cell number for each bacterial target. Samples with resultant CFU/mL of the considered probiotic bacteria over the fixed upper bound of 10^8^ CFU/mL per g of feces were excluded from the data processing, because the values exceed the maximum of the standard curve. For samples with Ct values around the lower bound of the detection limit, the corresponding CFU/mL were considered 10 (the minimum value obtained from the standard curve). Finally, if the Ct value was “undetermined”, 0 CFU/mL of that probiotic bacteria were considered in data analysis.

### 2.7. Gut Microbiome Community Characterization by Next Generation Sequencing

Fecal nucleic acid concentration and purity were estimated spectrophotometrically (NanoDrop™ One Microvolume UV-Vis Spectrophotometer, ThermoFisher Scientific, Rodano, Italy).

The amplification of the V3-V4 region of 16S rDNA was performed with Illumina MiSeq platform, employing v3 kit producing 300-bp paired-end sequences (that requires a load between 6 and 20 pM) at Biodiversa S.r.l., using 5 ng of NanoDrop quantified DNA as a template. The DNA sequence of interest was amplified using primer pair Pro341F (5′-CCTACGGGNBGCASCAG-3′) and Pro805R (5′-GACTACNVGGGTATCTAATCC-3′) [37]. All sequences were submitted to the European Nucleotide Archive (ENA) Database through the Bioproject N. PRJEB38178 with the name ena-STUDY-University of Milano-Bicocca-06-05-2020-00:03:05:422-646 (See Data Availability Paragraph).

### 2.8. Microbial Composition and Community Structure Analysis

To analyse the gut microbiota composition, Quantitative Insights Into Microbial Ecology 2 program (QIIME2, ver. 2019.4.) was employed. First, the raw paired-end FASTQ reads were uploaded, then a native plugin was used for the de-multiplexing step [38]. Subsequently, the Divisive Amplicon Denoising Algorithm 2 (DADA2) [39] was used for quality filtering, trimming, denoising, removing the mergepairs, and the chimeras. The ASV (Amplicon Sequence Variant) were assigned to taxonomic units employing the feature-classifier2 plugin [40] implemented in QIIME2 against the SILVA SSU non-redundant database (132 release), with a consensus confidence threshold of 0.6. A QIIME2 dedicated plugin was utilized for creating bar plots.

To assess the synbiotic intake effectiveness on the overall structure of the gut microbiota of the participants, the Faith phylogenetic index (Faith PD) was calculated [41].

To investigate if alpha diversity (Faith PD) changed significantly between the intervals of the three (successive/non-successive) different time-points, pairwise difference tests implemented in q2-longitudinal was employed [40].

Unweighted and weighted UniFrac distance were used to estimate community dissimilarity [42]. To establish possible connections between the observed microbial community variances and the synbiotic intake, non-metric multidimensional scaling analyses (NMDS), canonical correspondence analysis (CCA), analysis of similarities (ANOSIM), and multi response permutation procedure (MRPP) were applied.

Then, a “microbial maturity” index was computed with a regression model trained on feature data to quantify the relative rate of change over time in the three groups.

Indeed, the formulation of the Microbiota-by-Age Z-scores (MAZ) is calculated as Microbial maturity (MM)  =  predicted microbiota “age” (time-points) − median microbiota age of the control group (D) of a similar age (MAZ  =  MM/S.D.) as depicted by Subramanian et al. [43]. The results of the calculation are represented by a volatility plot, obtained comparing the relative “maturity” between each group [43]. The calculated MAZ scores were used as input metrics (dependent variables) in linear mixed effects models in order to test if considering the trend of the single ASV was possible to discriminate the three groups.

### 2.9. Statistical Methods

The Wilcoxon signed-rank test was used for pairwise comparison between time-points within a treatment group for qPCR data, as data were not normally distributed. The Mann-Whitney test was used for comparison between treatment groups, because data were not normally distributed. R version 3.5.1 was employed. Differences were considered statistically significant at *p*-value < 0.05 (*) and *p*-value < 0.01 (**).

The number of days related to CID symptoms was collected every day and is represented as the average number of days ± SEM. Potentially inappropriate stool or saliva samples were excluded from the analysis. Data regarding the markers of the immune system including fecal β-defensin2, salivary IgA, and salivary TAC were collected from each subject and are expressed as mean values ± SEM. Since data did not distribute normally, two tailed Mann-Whitney or Wilcoxon test were used for pairwise comparison [28]. R version 3.5.1 was employed. Statistically significant differences were considered as *p*-value < 0.05 (*) and *p*-value < 0.01 (**) [44,45].

Regarding fecal calprotectin, the number of subjects with marker levels below the normal value of 45 μg/g stool was recorded and the results were expressed as a percentage of subjects.

## 3. Results

### 3.1. Subjects of the Study

The study was conducted on a total of 75 Caucasian healthy elderly male and female subjects. The 75 enrolled subjects were randomly assigned to receive one mixture of prebiotic formulation (A group) or synbiotic formulation (B group) or placebo (D group) once daily (the first day was T0) for 28 days (T28); then the subjects attended 28 days of follow-up (T56) (Figure 1). The baseline characteristics of the volunteers of each group are presented in Table 1.

Concerning the age, we recorded a mean age of 70.08 ± 4.61 years in the synbiotic group, 68.76 ± 4.95 years in the prebiotic group, and 69.76 ± 5.20 years in the placebo group. Besides, the mean Body Mass Index (BMI) was 22.74 ± 1.38 Kg/m^2^ in the synbiotic group, 22.77 ± 1.68 Kg/m^2^ in the prebiotic group, and 23.25 ± 1.89 Kg/m^2^ in the placebo group. These characteristics were consistent with the specific inclusion criteria of the study. Among the treatment groups, demographic and benchmark features were comparable, denoting unbiased randomization.

All subjects included in the safety analysis dataset provided written informed consent before initiation of any study-related procedures. A questionnaire and an explanation of the protocol of the study were given to the subjects, including information about factors related to the modification of gut microbiota, such as the use of medications, the level of physical activity, tobacco use, and consumption of different food and supplement. Symptoms questionnaire was performed as an interview during the medical examination with the enrolled subjects at the time-points of the study.

### 3.2. Gut Microbiota Profile of Recruited Participants during the Treatment

**Figure 2:**
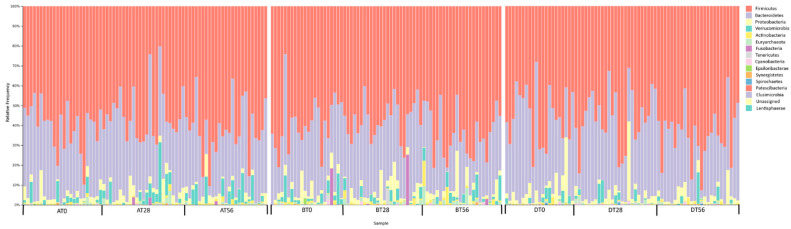
Gut microbiota composition of all the subjects analyzed by 16S rDNA V3-V4 region amplification at the three time-points (T0, T28, and T56). The relative abundance of bacterial ASVs at phylum level in every subject belonging to Prebiotic (A), Synbiotic (B), or Placebo (D) groups is shown by taxa bar plots. Each subject is identified by a bar and the different colored boxes represent the taxa (the percentage of relative abundance) within the sample. The legend shows the taxa from the most abundant (**left**) to the less abundant (**right**).

For each subject, fecal samples were collected, and DNA was extracted for the gut microbiota analysis. The V3-V4 region of 16S rDNA sequencing of the gut microbiota resulted in a total 24.952.554 reads. After DADA2 filtering step, we obtained a total of 16.813.759 reads with an average of 70.350 ± 57.098 reads for each sample, resulting in 14.839 ASVs [39]. A total of 16 phyla, 24 classes, 38 orders, 69 families, and 215 genera were identified (Table S4). Analysis of taxonomic profiles indicated a dominance of bacteria belonging to the following five phyla Firmicutes, Bacteroidetes, Proteobacteria, Verrucomicrobia, Actinobacteria. Figure 2 reports the relative abundances of these phyla in the analyzed datasets.

Moreover, considering the three groups in terms of relative abundances across the different time-points of the study, the most represented ASVs are assigned to *Bacteroides*, *Faecalibacterium*, *Prevotella*, *Alistipes*, *Subdoligranulum*, *Akkermansia*, *Roseburia*, *Blautia*, *Lachnospira*, and *Phascolarctobacterium* (Figure S1). In addition, although present in a no consistent manner, even the ASVs belonging to *Ruminococcus*, *Eubacterium*, *Veionella*, *Dorea*, *Butyricicoccus*, *Lactobacillus*, and *Bifidobacterium* were observed. Interestingly, the majority of the bacteria related to these ASVs positively influence the host gut metabolic processes.

**Figure 3:**
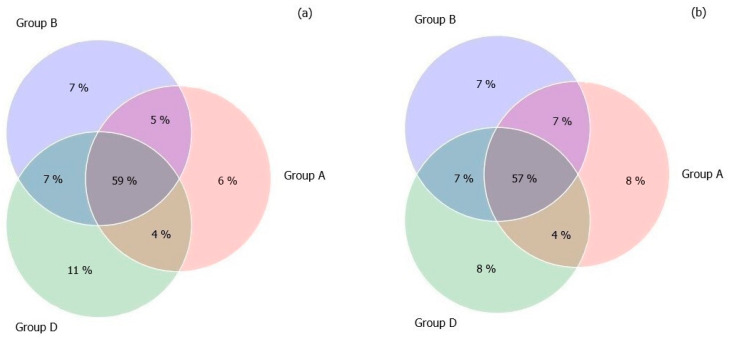
Venn diagram illustrating the percentage of Amplicon Sequence Variants (ASVs) that were shared or not shared by the gut microbiota of the three study-groups: Prebiotic (A), Synbiotic (B), or Placebo (D). Panel (**a**) represents the T28, and panel (**b**) represents the T56.

In order to evaluate the core microbiome of the three groups after the treatment (T28), we decided to compare the ASVs in a Venn Diagram. The results showed that 59% of ASVs were shared by the three groups, while the A, B, and D groups were dominated by ASV percentages of 6%, 7%, and 11%, respectively (Figure 3). A similar distribution was maintained at the end of the follow-up period (T56).

### 3.3. Gut Microbiome Diversity in Elderly Subjects

Bacterial diversity of the three groups (alpha diversity) was used to estimate the changes of biodiversity in the intervals of the three (successive/non-successive) time-points within each treatment-group. First of all, at the starting point (T0) (employing Mann-Whitney signed-rank test at two levels of significance) there was not a significant difference among the A, B, and D groups of the study. The pairwise difference test showed that there were no significant differences in Faith PD value in the interval between T0 and T28 for the three treatment groups. Both synbiotic (B group) and prebiotic (A group) treatments showed a significant increase between the initial and the final time-points (T0–T56) showing a *p*-value of 0.045 and 0.02, respectively. No significant increase was evidenced in the interval between T28 and T56, except for the placebo treatment (D group) (Table S5).

**Figure 4:**
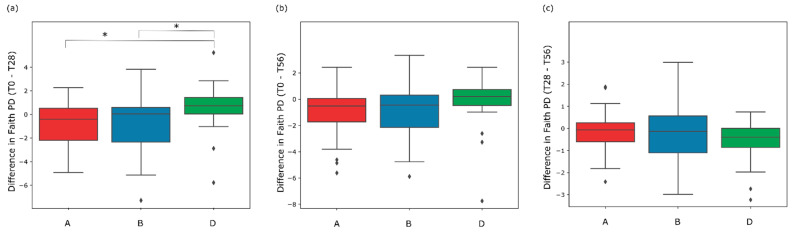
Comparison of the variation of the gut microbiota biodiversity by Faith PD value between Prebiotic (A), Synbiotic (B), and Placebo (D) groups during the following intervals of the study: the first 28 days of administration (T0–T28) (**a**), during the 56 days of the study (T0–T56) (**b**), and during the follow-up period (T28–T56) (**c**). Statistical differences between treatment groups were calculated using Mann-Whitney test (* *p*-value < 0.05).

In order to analyze the variation of the biodiversity among the three groups in the different time-points intervals, the pairwise group comparison test (employing Mann-Whitney test at two levels of significance) was applied. The Mann-Whitney test showed a significant variation between A *versus* D and B *versus* D groups after the administration period (T0–T28) (Figure 4a). Indeed, the boxplot showing the difference in Faith PD value of the interval T0–T28, highlights a significant difference of A and B groups with respect to D group (A vs. D U-value = 149 with *p*-value 0.02; B vs. D U-value = 301 with *p*-value 0.04). Considering the difference in Faith PD value between the baseline and the end of the follow-up (T0–T56), no significant difference was evidenced between A vs. D group and B vs. D (*p*-value > 0.05) (Figure 4b). Likewise, Figure 4c, representing the interval between T28 and T56, shows no significant difference from the comparison of A vs. D groups and B vs. D groups (*p*-value > 0.05). Overall, these results indicated that the synbiotic treatment led to a higher biodiversity variation after 28 days of treatment and the biodiversity is maintained until the end of the follow-up (T56).

**Figure 5:**
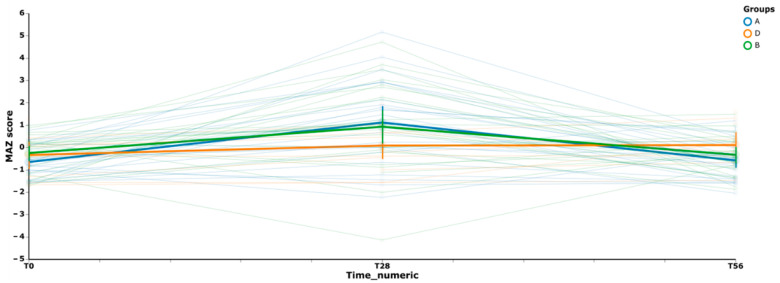
Volatility plot representing the trend of variation of the gut microbiota of the three treatment-groups. It represents the degree of change between selected time-points (T0, T28, and T56) and the resilience of the subject microbiota after the treatment with Prebiotics (A), Synbiotic (B), or Placebo (D). Thick curves are colored according to MAZ median-values, and the vertical bars represent error bars.

In order to thoroughly evaluate the variation of microbiota biodiversity between groups, the analysis of microbiota maturity (measured as MAZ score) was performed. We used a volatility graph (Figure 5) for assessing how MAZ score changed over time in each group. Figure 5, depicted as trend lines (representing the pattern observed for all the subjects of each group in the various time-points), shows a similar behaviour for both the prebiotic (A group) and the synbiotic (B group) treatments, while the placebo (D group) shows a different and stable trend.

**Figure 6:**
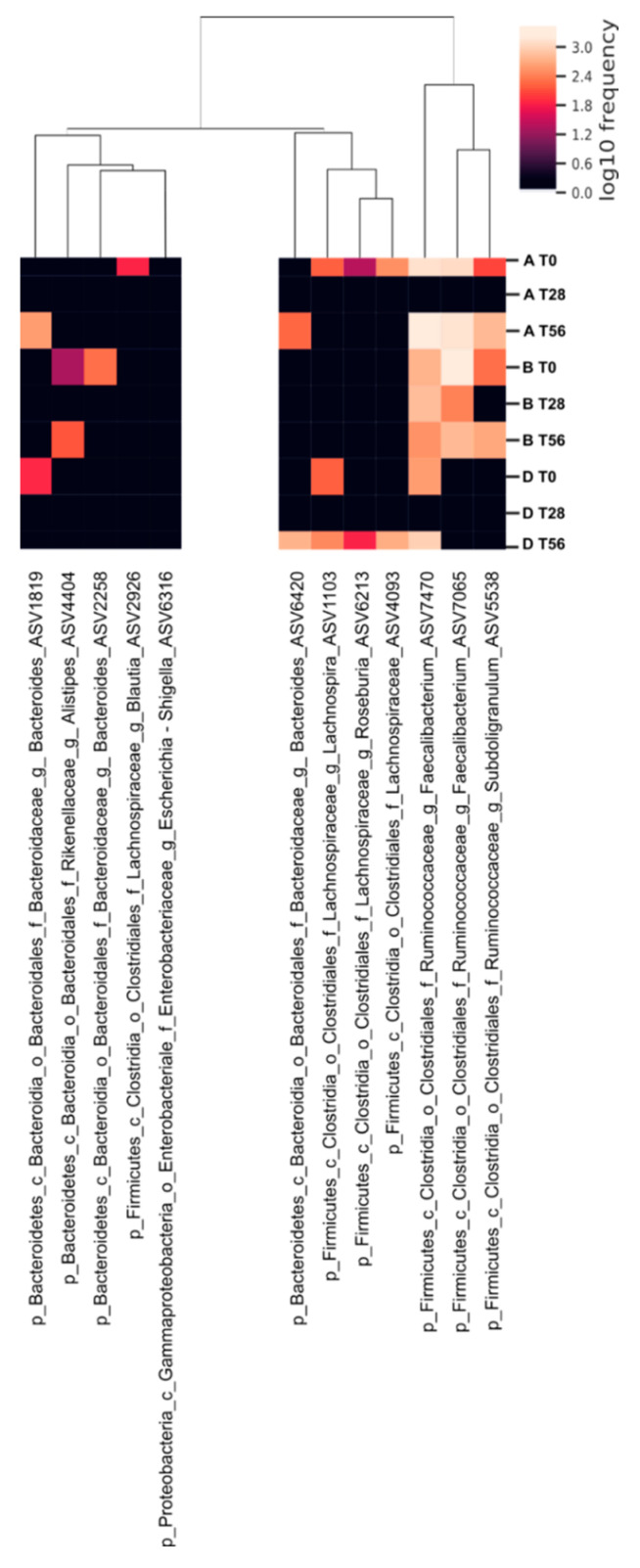
Heat map of ASVs variation of gut microbiota of elderly subjects of each treatment-group over time. In the logarithmic scale, color intensity indicates the abundance of each ASV feature. On the bottom of the heat map, the ASVs that varied for the three treatment-groups (Prebiotic A group, Synbiotic B group, Placebo D group) during the three time-points (T0 baseline, T28 time-point corresponding to the end of the treatment period, T56 time-point corresponding to the end of the follow-up period). On the top, the dendrogram of similarity between all the samples is represented.

The calculated MAZ scores indicated an increase of the community richness in A and B groups with respect to D group after the intake (T28). At the final time-point, there was a slight regression even if the MAZ score value remains higher in the synbiotic group compared to the prebiotic group. In general, the maturity analysis shows that the intake of synbiotic (B group) causes a variation in the structure of the microbial communities mainly at time T28. In addition, to appreciate the variation in microbiota biodiversity, a supervised learning regression was used to identify the most important ASVs able to promote a microbial shift in the treated groups across time. The analysis reveals that 50 different ASVs participated to discriminate the groups and that 12 ASVs belonging to *Bacteroides*, *Alistipes*, *Blautia*, *Lachnospira*, *Roseburia*, *Faecalibacterium*, and *Subdoligranulum* (*p*-value < 0.05) were the most abundant (Figure 6). In particular, among the ASVs that mostly contributed to the variation of the community biodiversity, the ASVs belonging to *Bacteroides* (ASVs 1819 and 6420) and *Subdoligranulum*_5538 showed a high variation in the A group at T56; while those belonging to *Blautia*_2926, *Lachnospira*_1103, *Lachnospiraceae*_4093, and *Roseburia*_6213 showed a lower frequency of variation at both T28 and T56. *Faecalibacterium* (ASVs 7470 and 7065) showed instability, reverting at time T56. The synbiotic treatment, instead, influenced *Faecalibacterium* (ASVs 7470 and 7065) to a highly stable frequency for all the duration of the study. Regarding the other ASVs mostly contributing to the variation in B group, we can observe that *Alistipes*_4404, *Bacteroides*_2258, and *Subdoligranulum*_5538 showed a frequency varying especially during the administration period. Concerning the ASVs frequency in the D group, we can affirm that no significant variation during the time of treatment was observed.

### 3.4. Effects of the Synbiotic Intake on the Most Relevant Taxa

**Figure 7:**
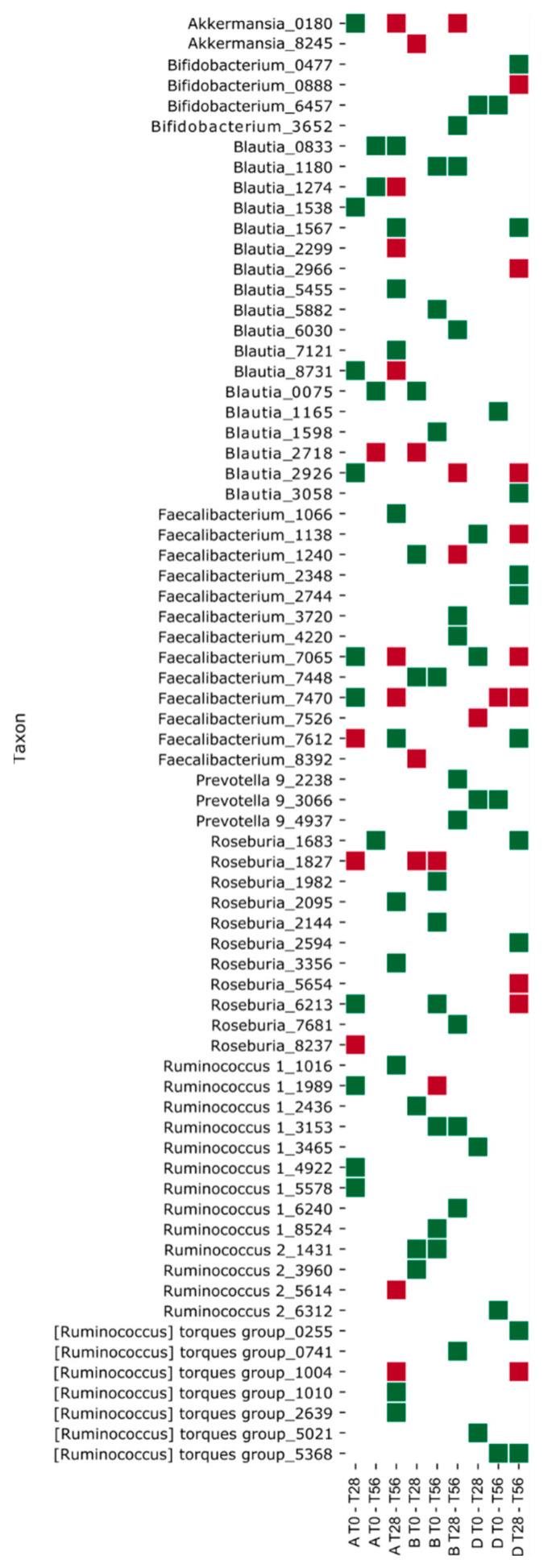
Heat map representing the significant biodiversity variation of ASVs assigned to the following bacterial genera *Akkermansia*, *Bifidobacterium*, *Blautia*, *Faecalibacterium*, *Prevotella*, *Roseburia*, and *Ruminococcus* for the three treatment-groups (Prebiotic A group, Synbiotic B group, and Placebo D group) during the three intervals of time-points (T0–T28 corresponding to the administration period, T0–T56 corresponding to 56 days of the study period, and T28–T56 corresponding to the follow-up period). Mann-Whitney test at two levels of significance was applied. The red squares depict a negative variation, the green ones depict a positive variation.

Often the bacteria with an immuno-modulatory role and producing several beneficial effects on the host are not the most abundant within the human intestinal microbiota [4]. In this context, an additional goal of the present work was the assessment of the synbiotic administration effectiveness on the intestinal microbiota taxa with a crucial role in gut metabolic functions. First, we evaluated the significant biodiversity variation of the gut microbiota community within synbiotic group with respect to the other two treatment-groups in the intervals of the three (successive/non-successive) time-points (employing Mann-Whitney test at two levels of significance). The ASVs that varied significantly are reported in Supplementary Materials (Figure S2). Among these, *Akkermansia*, *Bifidobacterium*, *Blautia*, *Faecalibacterium*, *Prevotella*, *Roseburia*, and *Ruminococcus* ASVs were considered for their high rate of variation and for their relevance in inducing gut metabolism changes [46] (Figure 7).

**Figure 8:**
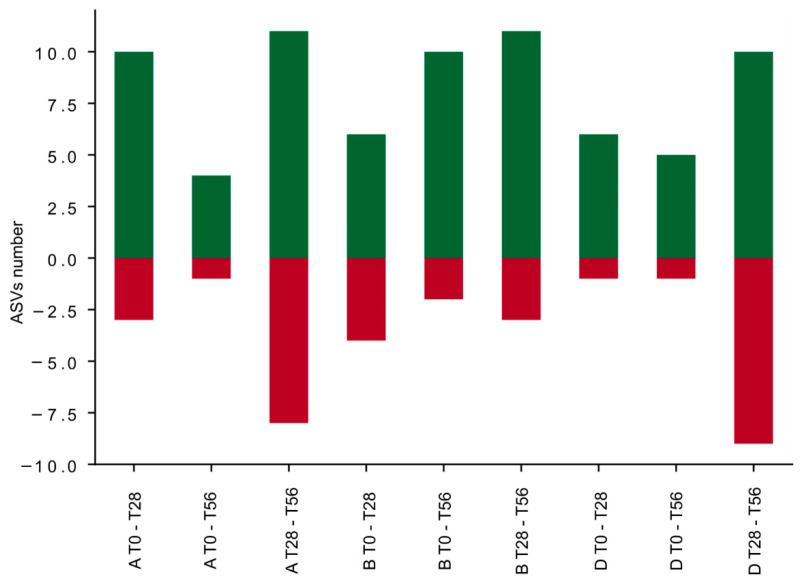
Bar plot representing the cumulative variation numbers of ASVs corresponding to seven bacterial genera *Akkermansia*, *Bifidobacterium*, *Blautia*, *Faecalibacterium*, *Prevotella*, *Roseburia*, and *Ruminococcus* for each treatment-group (Prebiotic A group, Synbiotic B group, and Placebo D group) during the three intervals of time-points (T0–T28 corresponding to the administration period, T0–T56 corresponding to 56 days of the study period, and T28–T56 corresponding to the follow-up period). The red squares depict a negative variation, the green ones depict a positive variation.

Summing the number of ASVs that showed a significant relative abundance variation at different intervals of time, we generated a differential bar plot. Positive cumulative variation (ASVs with positive abundance variation) is depicted with green bars and negative cumulative variation (ASVs with negative abundance variation) with red bars (Figure 8). The synbiotic treatment generated an increase of the ASVs number with a positive abundance variation (green bars) during all the intervals of time (T0–T28, T0–T56, and T28–T56) (Figure 8); and it generated a decrease of the ASVs number with a negative abundance variation (red bars). This can be appreciated by observing the specific positive variation (green squares) of the following ASVs: *Blautia*_0075, *Faecalibacterium*_1240, *Faecalibacterium*_7448, *Ruminococcus* 1_2436, *Ruminococcus* 2_1431, and *Ruminococcus* 2_3960 reported in the heat map of Figure 7 (T0–T28). During the follow-up period, the synbiotic treatment highlighted an increase of specific features (*Bifidobacterium*_3652, *Prevotella* 9_2238, and _4937) that in the prebiotic were not induced, although the placebo modulated diversely these two taxa. On the contrary, both the prebiotic and the placebo treatments induced a decrease of the ASVs number with a positive abundance variation during the two intervals T0–T28 and T0–T56. During the follow-up period (T28–T56), an increase of the ASVs number with a negative abundance variation is evidenced in the prebiotic and in the placebo groups. Nevertheless, the prebiotic intake induced a counterbalanced effect between T28 and T56, indeed the ASVs variation is comparable both in positive and in negative directions. Likewise, the D group demonstrated to have a similar response (Figure 8).

The assembled data could suggest that the synbiotic administration promotes a significant modulation of specific bacterial genera and/or combination of specific bacterial taxa of the gut microbiota contributing to the metabolic shifting, i.e., SCFA producers.

### 3.5. Effect of the Synbiotic on Lactobacillus *spp.* and Bifidobacterium *spp.*

**Figure 9:**
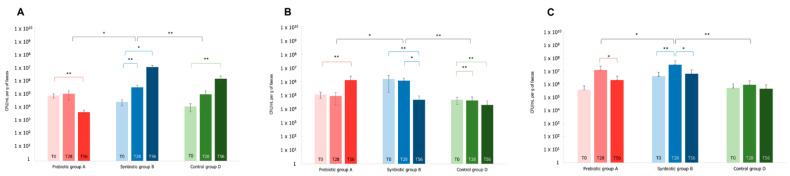
Quantification of cell numbers of *L. plantarum* (Panel (**A**)), *L. acidophilus* (Panel (**B**)), and *B. animalis* spp. *lactis* (Panel (**C**)) in fecal samples by species-specific qPCR at different time-points (T0 baseline, T28 time-point corresponding to the end of the treatment period, T56 time-point corresponding to the end of the follow-up period). Data are expressed as the mean values of CFU/mL per g of feces and error bars represent the SEM. The Wilcoxon rank-signed test was applied for the comparisons within each treatment group, highlighted in red, blue, and green color for Prebiotic (A), Synbiotic (B), and Placebo (D) groups, respectively. The Mann-Whitney test was applied for the comparisons between groups, reported in black color (* *p*-value < 0.05 and ** *p*-value < 0.01).

Commonly, the relative abundances of *Lactobacillus* and *Bifidobacterium* species within the intestinal microbiota are very low [4], particularly in the elderly people [11], although their beneficial properties. Considering these species, we observed around 30 and 70 ASVs belonging to *Lactobacillus* and *Bifidobacterium*, respectively. Interestingly, the number of *Bifidobacterium* ASVs increased after the synbiotic intake (T28), and a similar effect was evidenced by the prebiotic treatment towards both *Lactobacillus* and *Bifidobacterium* at T28. Instead, in the placebo group, a stable number of bacteria belonging to these genera with small physiological fluctuations was observed for the entire study. In order to specifically quantify the amount of bacteria belonging to the same species of the probiotics administered within the synbiotic i.e., *Lactiplantibacillus plantarum*, *Lactobacillus acidophilus*, and *Bifidobacterium animalis* subsp. *lactis*, the total DNA extracted from fecal samples (A, B, and D groups) was used to perform a qPCR analysis with species-specific primers (after sample collection at the times T0, T28, and T56). The qPCR analysis demonstrated that at time T0 in all the subjects of the three groups the average of the total cell amount of *L*. *plantarum*, *L*. *acidophilus*, and *B*. *lactis* was around 10^4^ CFU/mL g, 10^5^ CFU/mL g, and 10^6^ CFU/mL g, respectively (Figure 9).

In general, an increasing trend in the amount of these probiotic bacteria was observed in the treatment with the synbiotic (B group). Specifically, at time T28, a significantly higher amount of *L*. *plantarum* (*p*-value < 0.01) and *B*. *lactis* (*p*-value < 0.01) with respect to time T0 was evidenced in B group; furthermore, at the same time-point, *L*. *plantarum* and *B. lactis* were statistically higher compared to both A and D groups (*p*-value < 0.01: comparison of *L. plantarum* B vs. D, and *B*. *lactis* B vs. D; *p*-value < 0.05: comparison of *L. plantarum* A vs. B and *B. lactis* A vs. B, respectively) (Figure 9 panel A and C). The amount of *L*. *acidophilus* was stable and statistically higher with respect to both A (*p*-value < 0.05) and D (*p*-value < 0.01) groups at T28 (Figure 9 panel B). At time T56 different trends were detected. The amount of *L*. *plantarum* in B group was significantly higher, by two orders of magnitude compared to T28 (*p*-value < 0.05 T0 vs. T56). Instead, the amount of *L*. *acidophilus* and *B. lactis* decreased. Nonetheless, at T56 the total amount of *B*. *lactis* is comparable to the level of A group.

Overall, these results indicate that the synbiotic contributed to enhance the *Lactobacillus* and *Bifidobacterium* species within the gut microbial community after their ingestion for 28 days. Meanwhile, the persistence of the synbiotic strains in the gut system likely needs a longer period of administration.

### 3.6. Impact of Synbiotic Intake on Common Infectious Disease Incidence

The incidence of common infectious disease (CID) of the subjects was monitored during the entire study period. No subjects were drop-out or lost in the follow-up period and no adverse effects that are related to the administration of the treatments were recorded. The treatment efficacy in terms of CID symptom amelioration was evaluated both as the number of subjects that recorded at least one symptom of CID throughout the whole study (T0–T56), during the uptake period (T0–T28), and during the follow-up period (T28–T56), and as the average of days with one or more CID episodes.

Collected data highlighted that during both the uptake and the follow-up period (T0–T28 and T28–T56), B group experienced a minor percentage of subjects (36% and 12%) that had at least one CID episode with respect to A (48% and 20%) and D (56% and 20%) groups. Furthermore, considering the total period of the study (T0–T56), the same pattern was observed: the percentage of subjects that had at least one CID episode was minor in the synbiotic group (48%) with respect to placebo (76%) and prebiotic (68%) groups. Moreover, a lower percentage of subjects of B group presented the persistence of CID (20%) compared to the other two treated groups (A 24% and D 32%). Overall, these data indicated the synbiotic effectiveness in reducing the incidence of CID symptoms.

**Figure 10:**
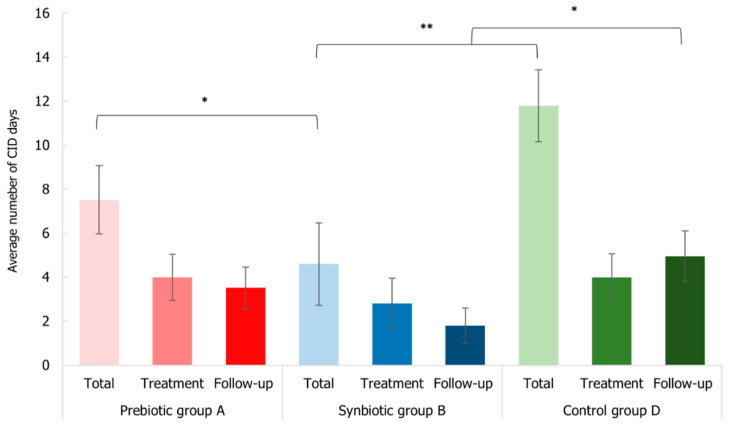
Average number of days related to common infectious disease (CID) symptoms that affected the elderly subjects at the different intervals of the three time-points (total period T0–T56, treatment period T0–T28, and follow-up period T28–T56) for Prebiotic (A), Synbiotic (B), and Placebo (D) groups. Data are expressed as mean ± SEM. The Mann–Whitney test was applied (* *p*-value < 0.05 and ** *p*-value < 0.01).

Considering the average number of days linked to CID, 5 ± 2 days was the average for B group (3 ± 1 during the administration period and 2 ± 1 during the follow-up period) and it was statistically lower than the average of the prebiotic (*p*-value < 0.05) and the control (*p*-value < 0.01) groups (Figure 10). Curiously, the average day number of the follow-up period of B group was significantly reduced (*p*-value < 0.05) with respect to D group, suggesting the persistence of the positive effect in the synbiotic group.

### 3.7. Immunological Markers Analysis

Since the gastrointestinal and the respiratory tracts are two interconnected systems, the immune reactivity was measured through the fecal calprotectin, the fecal β-defensin2, the salivary IgA, and the salivary TAC markers.

**Figure 11:**
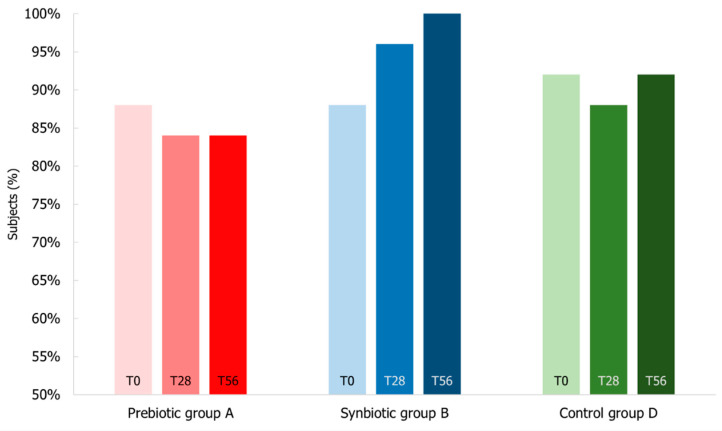
Percentage of subjects with levels of fecal calprotectin below the normal value of 45 µg/g. Levels were measured at T0 baseline, T28 time-point corresponding to the end of the treatment period, T56 time-point corresponding to the end of the follow-up period for Prebiotic (A), Synbiotic (B), and Placebo (D) groups.

Regarding fecal markers, Figure 11 shows the subject percentage presenting the fecal calprotectin level below 45 µg/g that is considered physiological for elderly people [29]. At the beginning of the study, 88% of the subjects of B group had normal levels of this fecal marker that after the synbiotic intake shifted in terms of number of subjects to 96% (T28) and 100% (T56) showing calprotectin level below 45 µg/g. Despite few unusual marker values, the calprotectin levels of prebiotic and placebo groups were stable during the study, presenting during the follow-up period 84% and 92% of subjects with calprotectin level below 45 µg/g, respectively.

**Figure 12:**
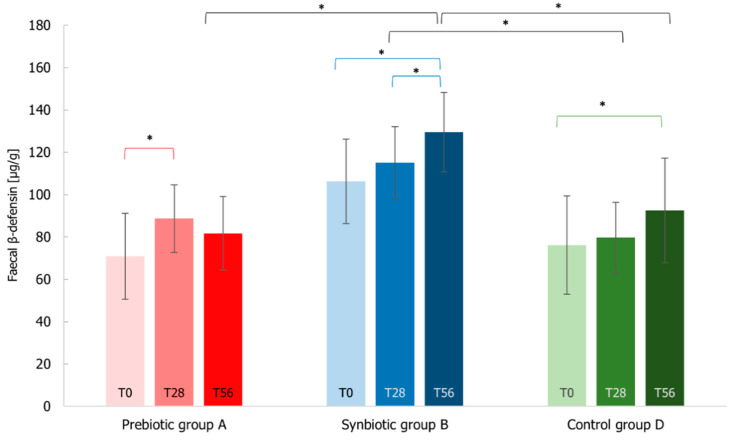
Fecal β-Defensin2 levels measured at T0 baseline, T28 time-point corresponding to the end of the treatment period, T56 time-point corresponding to the end of the follow-up period for Prebiotic (A), Synbiotic (B), and Placebo (D) groups. Values are expressed as mean ± SEM. The Wilcoxon rank-signed test was applied for the comparisons within each treatment group, highlighted in red, blue, and green color for Prebiotic (A), Synbiotic (B), and Placebo (D) groups, respectively. The Mann–Whitney test was applied for the comparisons between groups, reported in black color (* *p*-value < 0.05).

Data on the fecal β-defensin2 levels showed that the amount increased in all three groups (Figure 12). In particular, the synbiotic enhanced significantly (*p*-value < 0.05) the β-defensin2 levels with around 22% increase with respect to the beginning of the treatment (T0–T56). In addition, the levels of B group at the end of the follow-up period were statistically higher compared to both A (*p*-value < 0.05) and D (*p*-value < 0.05) groups. Although the subjects of the placebo group evidenced an increase at the end of the administration (*p*-value < 0.05), these values were not comparable to the ones of the synbiotic group. Even the prebiotic group presented a significant shift of these marker levels (*p*-value < 0.05) just at the first endpoint (25% increase with respect to the beginning of the treatment).

**Figure 13:**
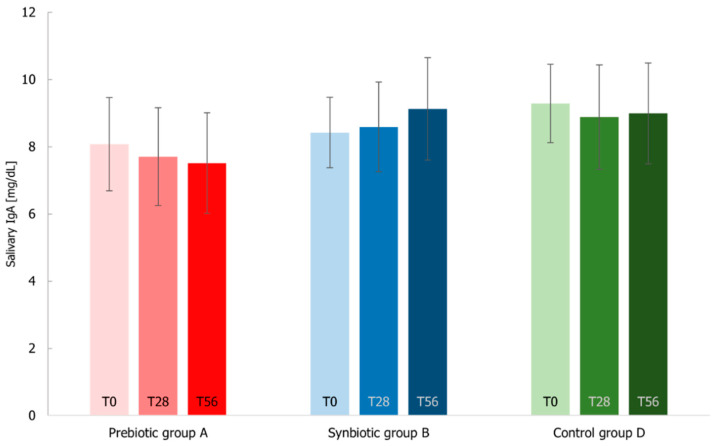
Salivary IgA levels measured at T0 baseline, T28 time-point corresponding to the end of the treatment period, T56 time-point corresponding to the end of the follow-up period for Prebiotic (A), Synbiotic (B), and Placebo (D) groups. Values are expressed as mean ± SEM. The Wilcoxon rank-signed test and the Mann–Whitney test were applied, and no significant differences were observed.

The elderly immune system associated with the respiratory tract is often characterized by the decrease of IgA and the increase of reactive oxygen species contributing to a state of senescence. Considering the salivary IgA values (Figure 13), the levels increased only for the synbiotic group (2% at T28 and 8% at T56 with respect to the beginning of the study, not in a significant manner), in line with the levels registered in elderly people [47]. For A and D groups, the levels decreased similarly at T28 (around 4.5%) and at T56 the prebiotic group decreased much more than the placebo group.

**Figure 14:**
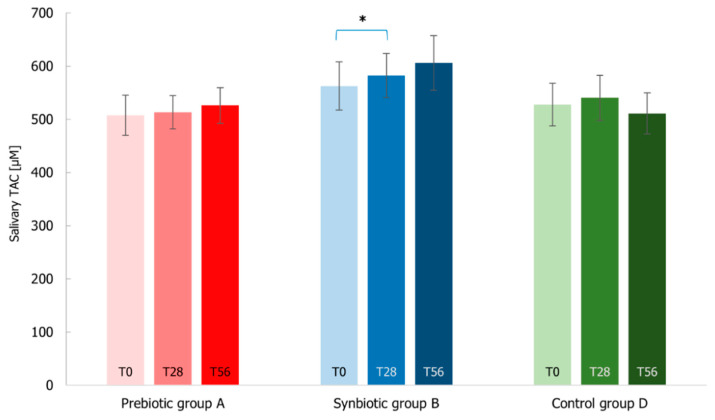
Salivary total antioxidant capacity (TAC) measured by FRAP assay at T0 baseline, T28 time-point corresponding to the end of the treatment period, T56 time-point corresponding to the end of the follow-up period for Prebiotic (A), Synbiotic (B), and Placebo (D) groups. Values are represented as mean ± SEM. Statistical differences were calculated using Wilcoxon rank-signed test (* *p*-value < 0.05). The statistical comparison within the synbiotic treatment group is highlighted in blue.

Finally, the TAC parameter measured by FRAP assay did not show important shifts after the treatments, except for the synbiotic intake that resulted in a significant increase of TAC levels between T0–T28 (*p*-value < 0.05) (Figure 14). Indeed, A and D groups presented stable TAC levels with an average of around 523 µmol considering both T28 and T56.

## 4. Discussion

Many studies report the health-promoting properties of probiotics or synbiotic formulations composed of *Lactobacillus* and *Bifidobacterium* strains in combination with prebiotics [22,23,48,49]. However, it is known that health properties are strain-dependent and prebiotic-dependent [4,25]. Probiotics elicit their functions through different ways, including the ecological effects on gut microbial communities and immune modulation mechanisms [25]. In addition, few probiotic/prebiotic synbiotic approaches have been reported considering the positive effect under the competitive conditions of the gastrointestinal tract [22]. In this context, we considered an experimental design of a clinical study including the synbiotic treatment (B group) with respect to the group with the prebiotics alone (A group) and with respect to the placebo group (D group). The clinical study was performed evaluating the effect of the new synbiotic on both the gut microbiota and the immune system of healthy elderly subjects. To our knowledge, no other study reports the elucidation of the synbiotic effects on both healthy and elderly population, associating the microbial gut composition with the markers of health-promoting properties, and including techniques that allowed the quantification of the probiotic strains. Moreover, in literature, the experimental design rarely comprised prebiotic alone and synbiotic intake. The overall data on the biodiversity of the intestinal microbial community collected in this work indicated that the synbiotic intake led to a higher biodiversity variation after 28 days of treatment with respect to the placebo group and the biodiversity was maintained until the end of the follow-up period (T56). Interestingly, the taxa that mostly contribute to the variation of biodiversity after the synbiotic treatment (B group) are different in comparison with the prebiotic alone (A group) during the time. This is in line with the majority of the literature data [48,50], in which the structure of the intestinal microbial community is principally modulated by the synbiotic intake [22]. Many papers report that the formulation efficacy in gut microbiota modulation addressed target taxa representing beneficial bacteria, including the increase of bifidobacteria, and promoting the decrease of a limited number of opportunistic pathogens and enterobacteria [12]. Moreover, in these studies, it is reported that the significant increase of probiotic strains (as lactobacilli and bifidobacteria) is induced by the fibers [4]. In our case, we observed a slight biodiversity variation of probiotic strains (as lactobacilli and bifidobacteria) probably due to the small amount of prebiotics administered to the subjects of A and B group, although the amount of the selected prebiotics was chosen to comply with the protocol of the clinical trial development; moreover, it is clearly established that autochthonous bacterial strains could metabolize the administered prebiotics starting an antagonistic relationship against probiotics for the same fiber utilization [23].

In any case, the analysis of the whole gut microbial community provided insights on the ecological and functional impact of the synbiotic administration, through the variation of biodiversity composition. After synbiotic administration (T0–T28), *Blautia*, *Faecalibacterium*, and *Ruminococcus* genera had a positive cumulative variation (Figure 8) that demonstrate the efficacy of the probiotic plus the prebiotics intake against the normal decrease in the gut of elderly subjects [48]. Moreover, during the follow-up period, the synbiotic administration highlighted an increase in the variation of specific features including *Bifidobacterium* and *Prevotella* that are associated with probiotic effect and fiber intake, respectively [4,51]. A similar change of bacteria profile in the gut microbiota in the elderly subjects after the synbiotic consumption was observed by Gao et al. and Costabile et al. [48,52]. These bacteria are considered beneficial gut microorganisms and candidates for the production of short-chain fatty acids (SCFA) [53], known for their positive effects on epithelial intestinal cells [54]. Interestingly, the observed ecological gut microbiota shift could indicate an amelioration in terms of metabolic functions. Indeed, *Faecalibacterium* taxa possess anti-inflammatory properties as well as actively contribute to the intestinal health and the maintenance of gut homeostasis [55], as does *Roseburia* genus [56]. *Akkermansia* is also known to promote immune modulation, healthy metabolic homeostasis, and protect against inflammation [46,57]. The presence of *Akkermansia* is often positively correlated with a high metagenome richness and specifically with *Ruminococcus* and *Bifidobacterium* taxa [58]. Additionally, *Faecalibacterium*, *Roseburia*, and *Ruminococcus* are considered as butyrate producing genera [4,22], *Blautia* and some species of *Akkermansia* are known as candidates for the SCFA production [53], whereas *Prevotella* genus is acknowledged as long-chain fatty acids producer [59].

Probiotic strains up-regulate the release of defensin molecules by the gut epithelium and favor the decrease of the fecal calprotectin of subjects with IBS or with visceral hypersensitivity [31,60]. As a primary outcome of our study, we analysed calprotectin and β-defensin2 as fecal biomarkers to follow inflammation in the gastrointestinal tract. Calprotectin is a calcium binding protein found principally in neutrophils and released in faeces upon accumulation and activation at the site of inflammation. It is also known that fecal calprotectin levels can change with age. Indeed, few healthy subjects of the study, especially belonging to B group, showed high level of calprotectin. Thus, the possible positive effect of the synbiotic treatment was slightly detectable. However, at the end of the study period (T56), all the subjects (100%) administered with the synbiotic exhibited normal calprotectin levels. On the contrary, in the presence of gut dysbiosis and disorders (IBS, Chrohn’s diseases, etc.), the levels of calprotectin usually are high and several times higher than the values of the healthy elderly at the end of our study [29]. Likewise, the increase of human β-defensin2 secretion was observed in healthy adult volunteers after administration of lactobacilli strains [61,62]. In these papers, the authors report that only a few strains favour the effect of the β-defensins, and they suppose that the difference among the strains is in the ability to induce the defensin expression correlated with the presence of genes encoding glycosylated cell surface structures. Our data confirmed literature data [31,48], suggesting a correlation between the effects on the microbiota of the subjects of B group and the immunological tested biomarkers with respect to the placebo group. In fact, we obtained an increase in the levels of the β-defensin2 and a reduction of the fecal calprotectin during the synbiotic treatment, associated with an increase in healthy microbiota in terms of diversity, stability, and resilience. Moreover, the positive stimulation of the innate gut immune system by the synbiotic treatment, for example, observed with β-defensin2 increase, can be associated with the intestinal cell reaction and the increase of beneficial molecule production by specific bacterial genera such as *Akkermansia*, *Prevotella*, and *Bifidobacterium*. This is further supported by literature, in fact, *Akkermansia* can exhibit potential anti-inflammatory responses and potentially modulates resident gut microbiota and together with *Bifidobacterium* are health-associated genera well-known for promoting immune modulation and protection against inflammation [57,58].

Because it is known that respiratory infections are dominant causes of diseases or mortality in the elderly for the association of aging and decline of the innate and adaptive immune system, we analyzed also salivary IgA and salivary total antioxidant capacity (TAC) for the respiratory tract. IgAs are important actors in the intestinal microbiota homeostasis preservation and in the defence against pathogens of gastrointestinal and respiratory tracts [15]. In our study, no significant responses deriving from the IgA markers were detected, except a slight increase after the synbiotic intake, as was also observed by Kotani et al. [13]. However, when the systemic inflammatory disorders increased for a different CID susceptibility in the subjects, the CID incidence was reduced after the treatment with the synbiotic formulate, similarly to the results Lefevre et al. [28], in which they observed a reduction of CID incidence in the elderly due to probiotic intake. Concerning the ability to counteract oxidative stress, such as the imbalance between ROS production and the defence of the organism, the TAC biomarker was monitored, suggesting a positive effect after the administration of the synbiotic formulation (B group) for the period of treatment. Indeed, this salivary homeostasis occurs as a result of aging that can be counterbalanced using prebiotic plus probiotic supplements [16].

In conclusion, our study strengthened literature data regarding the effects of a new synbiotic formulation on the gut microbiota biodiversity associated with biological markers of immune system (especially correlated to β-defensin2) with respect to the placebo treatment leading to an amelioration of the decline in the health status of the elderly. Indeed, to date, no literature data reporting specific immune parameters in elderly subjects are available. In fact, the most report only observational outcomes.

This study is characterized by several limitations. First, this study included a small number subjects, because of the difficulty of enrolling eligible elderly without severe concurrent diseases. A second criticism was the employing of immune biomarkers in the absence of clinical outcomes due to the difficulty of interpreting the biological significance of minor immunomodulatory effects [48]. Nevertheless, we chose calprotectin, β-defensin2, salivary IgA, and TAC biomarkers, because they are stable and ideal for non-hospitalized patients. Third, the minimum fiber concentration suggested for a functional synbiotic [63] was used in the study, that in theory could be sufficient to stimulate the cognate gut microorganisms. However, the synbiotic formulation was unconventionally combined with two *Lactobacillus* and one *Bifidobacterium* probiotics.

Overall, with the increased life expectancy worldwide, the development of strategies aimed to reduce the imbalance of the gut microbial community and to stimulate the immune system in the elderly is a challenging topic for nutraceuticals and probiotic supplementations. This study extensively demonstrates, from the gut microbiota to the clinical and metabolic point of view, that formulations with probiotics and prebiotics as nutritional supplementation can help to maintain a healthy status by re-equilibrating the gut microbiota of the elderly population.

## Figures and Tables

**Table 1:** Demographic data of the enrolled subjects. Results of Age and BMI are reported as mean values ± SD.

	A Group	B Group	D Group
**N° of subjects (female/male)**	25 (20/5)	25 (20/5)	25 (17/8)
**Age (years)**	68.76 ± 4.95	70.08 ± 4.61	69.76 ± 5.20
**BMI (kg/m^2^)**	22.77 ± 1.68	22.74 ± 1.38	23.25 ± 1.89

## Data Availability

The 16S rDNA sequences generated in this study have been deposited into ENA Database. The corresponding Metadata information is provided as Table S4 in the Supplementary Materials.

## Supplementary Materials

The following supporting information can be downloaded at: https://www.mdpi.com/article/10.3390/microorganisms10061256/s1, Table S1: List of the lactic acid bacteria strains used in this study, deposit number and their most relevant antimicrobial characteristics are described in Presti et al. and De Giani et al. [25,26]. Table S2: Composition and characteristics of prebiotics in the administered formulations used in this study. Table S3: List of the species-specific primers used in this study for qPCR analysis (adjusted from Mezzasalma et al. [27]). Table S4: Metadata information of 16S rDNA sequences. The sequences that were generated in this study have been deposited into the EMBL-EBI Database. Table S5: Pairwise difference test on the alpha diversity of A, B, and D groups in the different intervals of time (T0–T28, T0–T56, and T28–T56) using Wilcoxon signed-rank test. Figure S1: Gut microbiota composition of all the subjects analyzed by 16S rDNA V3-V4 region amplification at the three time-points (T0, T28, and T56). The relative abundance of bacterial ASVs in every subject of Prebiotic (A), Synbiotic (B), or Placebo (D) groups is shown by taxa bar plots. Each subject is identified by a bar and the different colored boxes represent the taxa. The order from the top to the bottom of each ASVs at genus level corresponding to a different colored box represents the percentage of relative abundance of bacteria within the sample. Figure S2: Heat map of ASVs variation in gut microbiota biodiversity of elderly subjects of each treatment-group over time. Green color indicates the highest abundance of each ASV feature, red color the lowest. On the bottom of the heat map, the ASVs that varied for the three treatment-groups (Prebiotic A group, Synbiotic B group, Placebo D group) during the three intervals of time-points (T0–T28 corresponding to the administration period, T0–T56 corresponding to 56 days of the study period, and T28–T56 corresponding to the follow-up period).
