# Supplementary material for: Effects of Inulin-Based Prebiotics Alone or in Combination with Probiotics on Human Gut Microbiota and Markers of Immune System: A Randomized, Double-Blind, Placebo-Controlled Study in Healthy Subjects
Source: Microorganisms. 2022 Jun 20;10(6):1256. doi: 10.3390/microorganisms10061256 (PMC9229734; doi:10.3390/microorganisms10061256)
Supplement: Supplementary file 1 [file microorganisms-10-01256-s001.zip › microorganisms-1758040-supplementary/Table S2_Front.pdf]

Table S2. Composition and characteristics of prebiotics in the administered formulations used in this study.

Fiber selected for the study	Description
FOS	50 mg of fructo-oligosaccharides with a degree of polymerization between 3-5
Inulin 90%	50 mg of inulin from chicory with a degree of polymerization of 10
