# Supplementary material for: Effects of Inulin-Based Prebiotics Alone or in Combination with Probiotics on Human Gut Microbiota and Markers of Immune System: A Randomized, Double-Blind, Placebo-Controlled Study in Healthy Subjects
Source: Microorganisms. 2022 Jun 20;10(6):1256. doi: 10.3390/microorganisms10061256 (PMC9229734; doi:10.3390/microorganisms10061256)
Supplement: Supplementary file 1 [file microorganisms-10-01256-s001.zip › microorganisms-1758040-supplementary/Table S3_Front.pdf]

Table S3 List of the species-specific primers used in this study for qPCR analysis (adjusted from Mezzasalma et al., 2015).

Probiotic	Primer code	Sequence (5'→ 3')	DNA region	Amplified length (bp)
L. plantarum	Lpl2F<br>LpL2R	CATTGGAACCGAACCAGTTG<br>CGGTGTTCTCGGTTTCATTATG	16S/23S IS	203 bp
L. acidophilus	Lacid2F<br>Lacid2R	GGGCAAATCACGAACGAGTA<br>CTTTGTTTTCGTTCGCTTCA	pre16S	132 bp
B. animalis subp. *lactis*	AnimF<br>AnimR	GCACGGTTTTGTGGCTGG<br>GACCTGGGGGACACACTG	pre 16S	171 bp
