# Supplementary material for: Effects of Inulin-Based Prebiotics Alone or in Combination with Probiotics on Human Gut Microbiota and Markers of Immune System: A Randomized, Double-Blind, Placebo-Controlled Study in Healthy Subjects
Source: Microorganisms. 2022 Jun 20;10(6):1256. doi: 10.3390/microorganisms10061256 (PMC9229734; doi:10.3390/microorganisms10061256)
Supplement: Supplementary file 1 [file microorganisms-10-01256-s001.zip › microorganisms-1758040-supplementary/Table S5_Front.pdf]

Table S5 Pairwise difference test on the *alpha*-diversity of A, B, and D groups in the different intervals of time (T0 - T28, T0 - T56, and T28 - T56) using Wilcoxon signed-rank test.

Group	W (Wilcoxon signed-rank test)	p-value	FDR p-value
**T0 - T28**			
A	93.0	0.103	0.155
B	81.0	0.230	0.230
D	66.0	0.085	0.155
**T0 - T56**			
A	69.0	0.020	0.061
B	58.0	0.045	0.068
D	100.0	0.590	0.590
**T28 - T56**			
A	136.0	0.476	0.692
B	125.0	0.692	0.692
D	50.0	0.002	0.007
