# Supplementary material for: Effects of Inulin-Based Prebiotics Alone or in Combination with Probiotics on Human Gut Microbiota and Markers of Immune System: A Randomized, Double-Blind, Placebo-Controlled Study in Healthy Subjects
Source: Microorganisms. 2022 Jun 20;10(6):1256. doi: 10.3390/microorganisms10061256 (PMC9229734; doi:10.3390/microorganisms10061256)
Supplement: Supplementary file 1 [file microorganisms-10-01256-s001.zip › microorganisms-1758040-supplementary/Table S1_Front.pdf]

Table S1 List of the lactic acid bacteria strains used in this study, deposit number and their most relevant antimicrobial characteristics are described in Presti et al. 2015 and De Giani et al. 2019.

**Probiotic strain**	**Deposit number**	**Antimicrobial activity *vs***	**Growth capacity on inulin-type fructans (different Degree of Polimerization DP)**
Lactiplantibacillus plantarum (formerly *Lactobacillus plantarum)* PBS067	DSM 24937	*C. albicans; E. faecalis; P. aeruginosa; S. aureus; E. coli.* Production of bacteriocin-like compound (P1053) active against *S. aureus* and *E. coli*	FOS DP 3~5; FOS DP~10; Inulin DP~25
Lactobacillus acidophilus PBS066	DSM 24936	*C. albicans; E. faecalis; P. aeruginosa; S. aureus; E. coli*	FOS DP 3~5
Bifidobacterium animalis subsp. *lactis* BL050 (formerly PBS075)	DSM 25566	*E. faecalis; P. aeruginosa; E. coli*	FOS DP 3~5; FOS DP~10; Inulin DP~25
